# The Throat Department at St. Mary's Hospital

**Published:** 1889-07-06

**Authors:** 


### The Hospital Workshop.

### No. IV.—THE THROAT DEPARTMENT AT ST. MARY'S HOSPITAL.

(BY OUR SPECIAL COMMISSIONER.)

There is something about the building of St. Mary's which shows the architect has had to make the most of his ground-space. One is reminded of the old cities within walls where people delved deep in the earth, and stretched their houses skyward in their search for more room. The out-patient wing everywhere discloses this cunning of construction. Patients seem to dive from the street in some mysterious way into a kind of dim ante-chamber, where attendance-books are given out. From thence opens a lofty waiting-hall, on one side of which are placed the consulting-rooms, two sets on the ground level, and four others upstairs. Ventilation is good, and the walls are lined with glazed white brick, a capital plan, that combines light with cleanliness. Further details will be dealt with in a subsequent paper. Meanwhile let us see something of the work that is going on inside, and send in a card by the porter, choosing at random the right-hand room on the main floor. This is Friday, and chance has guided our visit to the throat department. The physician in charge, Dr. Scanes Spicer, received your commissioner courteously, and during the next couple of hours imparted much valuable information, from which we shall select such portions as seem likely to be of interest to general readers.

The first case is that of a highly-nervous girl of eighteen. She is pale and shaky, and cannot control the muscular twitchings that take place in her hands and elsewhere. Indeed, these grow worse when her attention is called to them, and suggest a mild St. Vitus dance. What has brought her here, however, is a "choky" feeling and a "lump" in the throat. External examination shows a curious growth known as an "accessory thyroid"—that is to say, attached to the gland that lies in front of the windpipe just below "Adam's apple" is an offshoot about the size of a walnut, the pressure from which may perhaps account for the symptoms. It may seem a curious statement that irritation about the nose or throat should be the starting-point of nerve-storms like St. Vitus dance, yet beyond a doubt, that is what sometimes happens. Later on, this matter will be more fully referred to, when speaking of reflex diseases caused in the same way. The girl is ordered tonics, diet, and general treatment. She is followed by a medical student, who complains of sore throat and difficulty of swallowing. The tonsils are greatly enlarged, and a glance tells one that the mischief has been there for some time. This is remarkable, as the patient is a long-distance runner, who of all men would require his breathing free and unhampered. Removal of the tonsil is recommended by the physician, but not until the surrounding inflammation has been subdued by a course of sprays and gargles. At the same time, a careful examination is made of the nose, as it is very frequently the source of throat troubles.

Here is a man of sixty-nine, wrapped up in woollen scarves, who has been sent from the country by his medical attendant, an old St. Mary's student. By trade the patient is a builder. He tells us that lately he has been losing flesh, and for the last four months has had an increasing difficulty in swallowing. Now he can take fluids and soft food only. On feeling from the outside the throat is hard as stone, very much as if the cartilages had become bony, a change which occurs now and then in advanced age. A little mirror is warmed and passed to the back of the throat, so as to reflect the "larynx" or cavity at the entrance of the windpipe. This instrument is the "laryngoscope," which has laid the foundation of the modern science and art of throat surgery. In the present case it shows that the vocal cords are ulcerated, but the exact nature of the affection is by no means clear. Dr. Spicer passes a "bougie"—which is a light pliable rod of gum-elastic—down the gullet, but it meets with no hindrance, showing that the disease is most probably confined to the air passage. The old man says he has passed a healthy open-air life, and admits having lived freely. He has with him an officious friend, who all along insists on putting in remarks that have little or no bearing on the case. Thus, when the patient was questioned as to his voice, the talkative ally at once volunteered, "No, he don't sing. I never 'eard 'im sing anything in my life—not right through, iike—nothing more than a snatch. And he don't smile—only very rarely—and don't never laugh: he never wor that way inclined." And then he wandered on into further details, of which we catch something about a "drop of Scotch whisky mixed with summut in the shape of a hegg or two." At the same time, he is really careful of the poor old builder, who is in a bad way, although it is a kind of politeness that somehow suggests a public-house and a sanded floor. So far as the throat is concerned, if the condition gets worse the patient will have to be fed by a soft indiarubber tube passed into the stomach and fitted to a funnel. Not many years ago, if the gullet were quite obstructed, it meant certain death to the person so afflicted. But surgeons nowadays feed the sufferer somewhat after the fashion of a mollusc, through an opening made into the stomach, using artificially digested food for the purpose.

Next comes a mother who says her little child snores very much at night, and has something wrong with its throat. When children sleep with their mouths wide open, and breathe noisily, enlargement of the tonsils is generally present, in many cases as a result of nasal irritation. Very commonly in the space that lies at the back of nostrils and above the throat are a number of growths like tiny warts. Our little patient suffers from these, and also from swollen tonsils. These curious growths are called "adenoids" (*i.e.*, "gland-like," because of their structure), and are sometimes found in the larynx, the cavity already referred to at the entrance of the windpipe, which contains the vocal cords, and lies just behind "Adam's apple." They are best removed from the back of the nose by scraping with the finger-nail, and from the larynx by a curved forceps. Now and then they grow all through life in immense numbers. A case of this kind is reported where a patient was first operated on at the age of twelve. This was followed by four or five hundred different operations spreading over many years. In time the patient grew so learned that he was able to use the laryngeal mirror and clear away for himself the growths that crowded his larynx and vocal cords.

Persons occasionally come to the hospital with a foreign body, such as a fruit stone or a bone, lodged in the larynx, where it leads to much distress, or even suffocation. An extremely interesting case is on record. A peddler swallowed the "Brummagem" earring he was trying to pass off as gold. His determination and presence of mind, however, did not prevent him getting sentenced to six months' imprisonment, possibly because the unswallowed earring was still in evidence. While in gaol he complained of pain in the throat, great discomfort, hoarseness, and cough, and having no object for further concealment, confessed to what he had done to escape detection. The laryngeal mirror showed the ornament fixed in his windpipe, and, although strongly urged to have an opening made lower down, he would not submit to this operation of "tracheotomy." After some time he was seized with a sudden fit of distressed breathing, along with pain in the chest and spitting of blood, symptoms which showed that the foreign body had slipped down into one of the bronchial tubes. The unfortunate man was turned upside down and shaken, while a surgeon stood by ready to open the throat in case of need. At the time these efforts did not succeed in dislodging the earring, but an hour afterwards it was coughed up by the patient. Mr. Bernard Pitts, of St. Thomas's Hospital, had the case under treatment and shows the two earrings side by side, the one still spick and span and seemingly of fine gold, while the other is stained and shabby from its wanderings in the air-passages of the unlucky peddler.

Specialism is making vast strides in every branch of medical science, and its achievements and possibilities are strikingly shown in the study of the nose and throat. The subject is full of interest. For instance, snoring is shown in many cases—as in that of the child already mentioned—to depend upon some blocking of the nostrils. So that parents of a future and wiser age, instead of reproaching their children for an unbecoming habit, will take them off to consult a specialist. But more extraordinary still are the reflex diseases traceable to irritation within the nose. Readers may be reminded that a reflex nervous action occurs when the stimulus applied to a sensory nerve-ending is carried to a centre and thence reflected through a motor nerve, causing muscular movements in various parts of the body. For instance, a person takes a pinch of snuff, and the nasal nerves convey the stimulus to the brain, where it is sent out again through the respiratory nerves, and a strong blast of air or "sneeze" is directed through the nostrils, with the object of driving away the offending particles. In the place of snuff, imagine the inflamed mucous membrane of a common "cold in the head," and sneezing again results. It is now proved that other unhealthy conditions of the nose are capable of setting up more or less violent reflex actions in parts away from the source of the mischief. Among these may be mentioned headaches, St. Vitus dance, and asthma. In a certain number of cases asthma can be traced to nasal irritation, which, by reflection through the brain, causes the spasmodic closure of the bronchial tubes to which that affection is due. Physicians have long known this distressing malady commonly springs from reflex irritation, but it is often impossible to make out exactly where. Now comes a practical point. If the exciting cause can be discovered and remedied before the disease has become, so to speak, a fixed habit, then recovery may be confidently looked for. Some observers trace epilepsy to the same source. Of course, nasal irritation will not account for more than a small proportion of cases. Nevertheless, each one thus brought under control is a laurel added to the crown of progressive specialism. In conclusion, it may be remarked that the state of the nasal cavity affords a key to many affections of the ear, the throat, and the lungs. The reasons for this are various, such as spread of inflammation, irritation of altered excretions, reflex disturbance, and upsetting of function generally. Hence the specialist for the throat and ear always makes a point of examining the nose.

Your commissioner is pleased to add that Dr. Spicer recommended a patient to take her child home and call in a neighbouring medical man. It would help to settle the grievances of the general practitioner against the hospitals were such a course more often adopted. Those cases only which are obviously unfitted either because of the patient's position or from the nature of the ailment, would be rejected. Moreover, physicians and surgeons who are not interested in the mere swelling of attendance lists, would thereby diminish the burden of over-crowded waiting-rooms, and at the same time lessen their own drudgery.

